# Middle meningeal artery embolization for chronic subdural hematoma refractory to Burr hole surgery: A case report

**DOI:** 10.1002/ccr3.8812

**Published:** 2024-05-06

**Authors:** Subash Phuyal, Sushanta Paudel, Suchit Thapa Chhetri, Gopal Sedain, Prakash Phuyal, Oshan Shrestha, Deepa Khanal

**Affiliations:** ^1^ Department of Neuroradiology Upendra Devkota Memorial National Institute of Neurology and Allied Sciences Kathmandu Nepal; ^2^ Nepalese Army Institute of Health Sciences Kathmandu Nepal; ^3^ Department of Neurosurgery Tribhuvan University Teaching Hospital Kathmandu Nepal; ^4^ Manipal College of Medical Sciences Pokhara Nepal

**Keywords:** Burr hole surgery, chronic subdural hematoma, endovascular procedure, middle meningeal artery embolization

## Abstract

Middle meningeal artery embolization is a valuable alternative for chronic subdural hematoma refractory to Burr hole surgery. In a 61‐year‐old patient, this endovascular intervention effectively resolved the hematoma alleviating associated symptoms.

## Graphical abstract

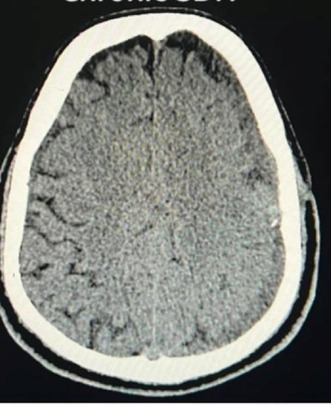

## 1 INTRODUCTION

Chronic subdural hematoma (CSDH) commonly results from minor trauma, leading to the rupture of cortical bridge veins and subsequent recurrent microhemorrhages.^1^ Representing a complex spectrum of pathology, untreated chronic subdural collections can pose significant morbidity, and mortality risks. The occurrence of CSDH is approximately 1.72 cases per 100,000 people annually, but it significantly increases to 7.35 cases per 100,000 per year within the age range of 70–79 years.^2^

CSDHs manifest as collections of fluid, blood and blood degradation products between the dura mater and the arachnoid mater.^3^ Local inflammation is believed to be a contributing factor to CSDH formation, leading to hyperfibrinolysis of the clot.^4^, ^5^, ^6^ This process results in the production of angiogenic factors, inducing neovascularization, and bleeding from fragile capillaries.^5^, ^7^, ^8^ The middle meningeal artery plays an important role in the development and treatment of recurrent CSDH.

Here we present a case of CSDH refractory to Burr hole surgery managed endovascularly by embolization of the middle meningeal artery.

## 2 CASE REPORT

A 61‐year‐old male with a medical history of Hypertension and Type II Diabetes Mellitus presented to the emergency department reporting a sudden onset of headache and a single episode of vomiting. The headache, localized in the frontoparietal region, was described as progressively dull and aching, nonradiating, without specific aggravating or relieving factors associated with the vomiting. The patient denied any family history of similar illnesses, and there was no reported trauma, loss of consciousness, slurring of speech, or abnormal body movements.

### 2.1 Timeline

**FIGURE 1:**
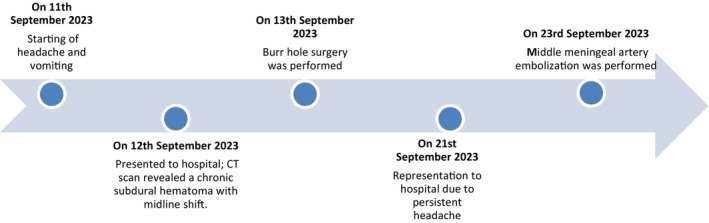
Timeline showing major events.

The chronological sequence of major clinical events is presented in the following timeline in Figure 1.

### 2.2 Diagnostic assessments

**FIGURE 2:**
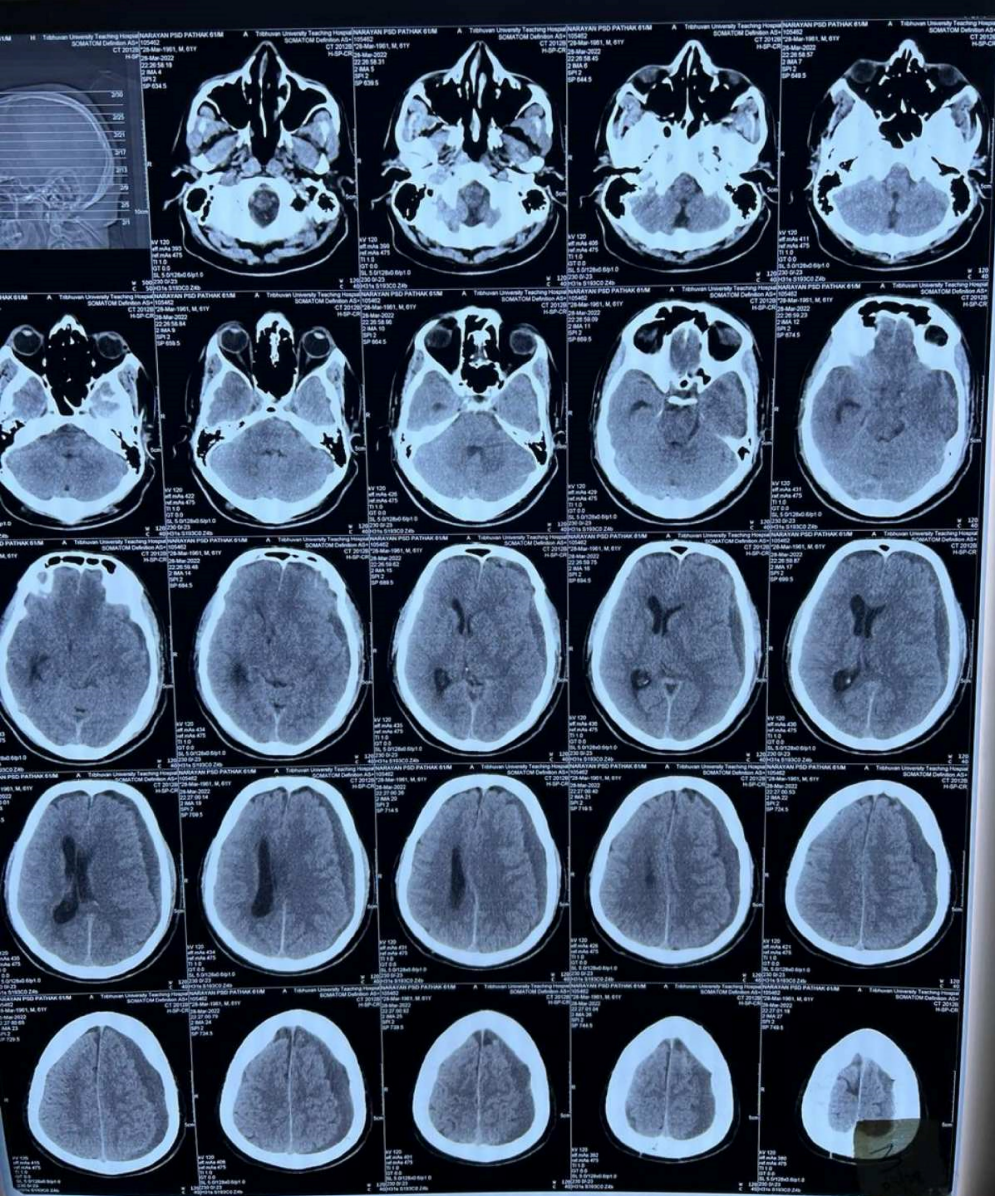
CT scan showing accumulation of blood in the subdural space with midline shift during the initial presentation.

Upon examination, the patient exhibited a Glasgow Coma Scale score of 15/15, intact cranial nerves, no focal deficits, and no signs of meningism. Bilateral grade II deep tendon reflexes with a downgoing plantar reflex were noted. Pupils were 2 mm in diameter bilaterally and reactive to light. There were no other significant neurological findings. The patient's blood pressure was recorded at 156/96 mm of Hg, pulse at 80 bpm, respiratory rate at 22/min, and oxygen saturation at 96 mm Hg. Upon laboratory investigations, the calcium (8.4 mg%) and magnesium (1.6 mg%) levels were borderline. The important laboratory findings are detailed in Table 1. Serology tests for HBsAg, HCV, HIV I and II, and SARS‐CoV antigen were negative. A Computed Tomography (CT) scan revealed a CSDH with midline shift. (Figure 2).

**TABLE 1:** Laboratory findings of patient on presentation.

Laboratory investigations	Values
Hemoglobin	13.6 gm%
Sodium	142 mEq/L
Potassium	3.2 mEq/L
Random blood glucose	88 mg/dL
Calcium	8.4 mg%
Magnesium	1.6 mg%

### 2.3 Treatment

**FIGURE 3:**
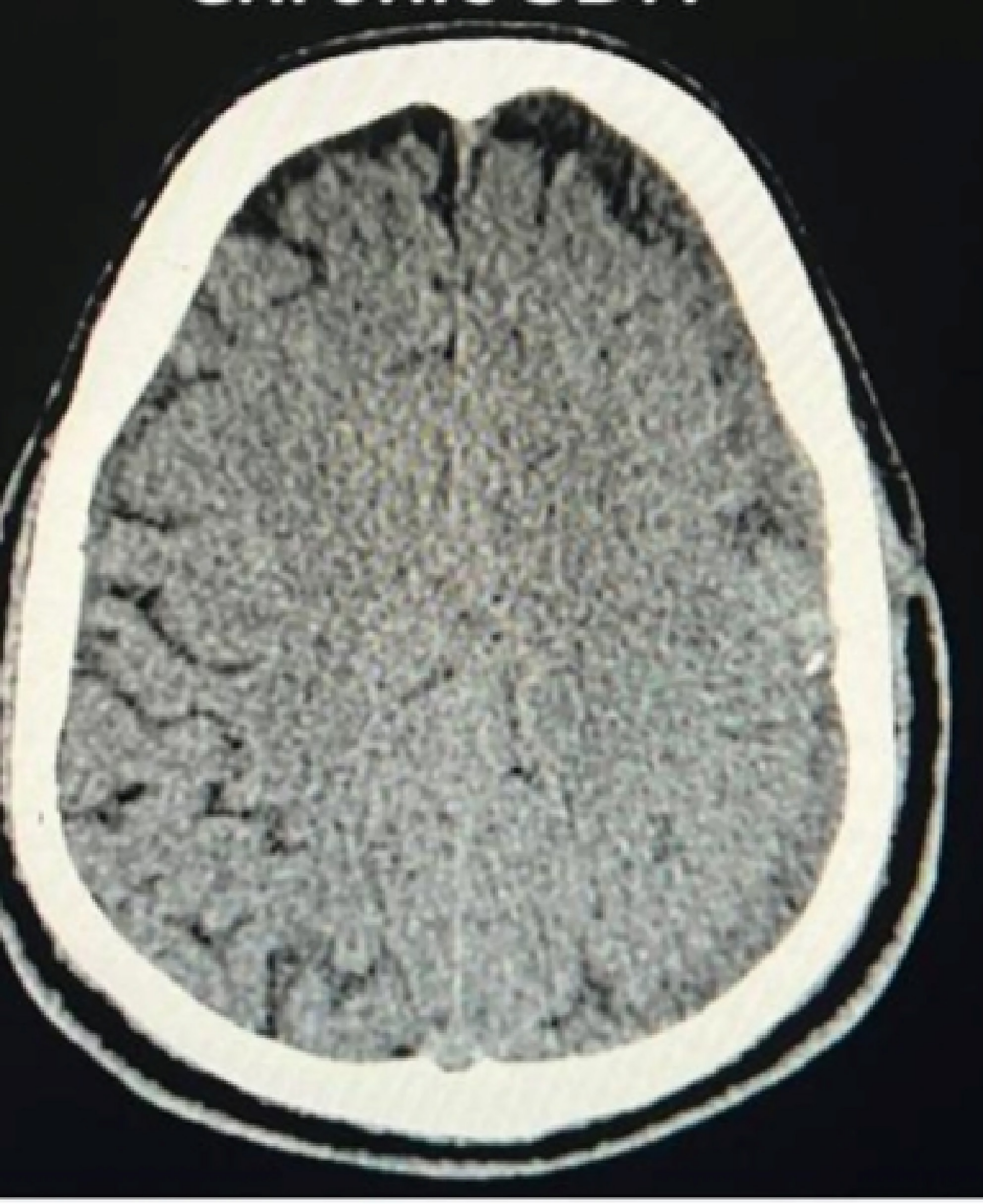
CT scan showing reaccumulation of blood in the subdural space after burr hole surgery.

The patient underwent Burr hole surgery for hematoma evacuation. Despite surgery, persistent headache prompted a postoperative CT scan, revealing reaccumulation of blood in the subdural space. (Figure 3).

**FIGURE 4:**
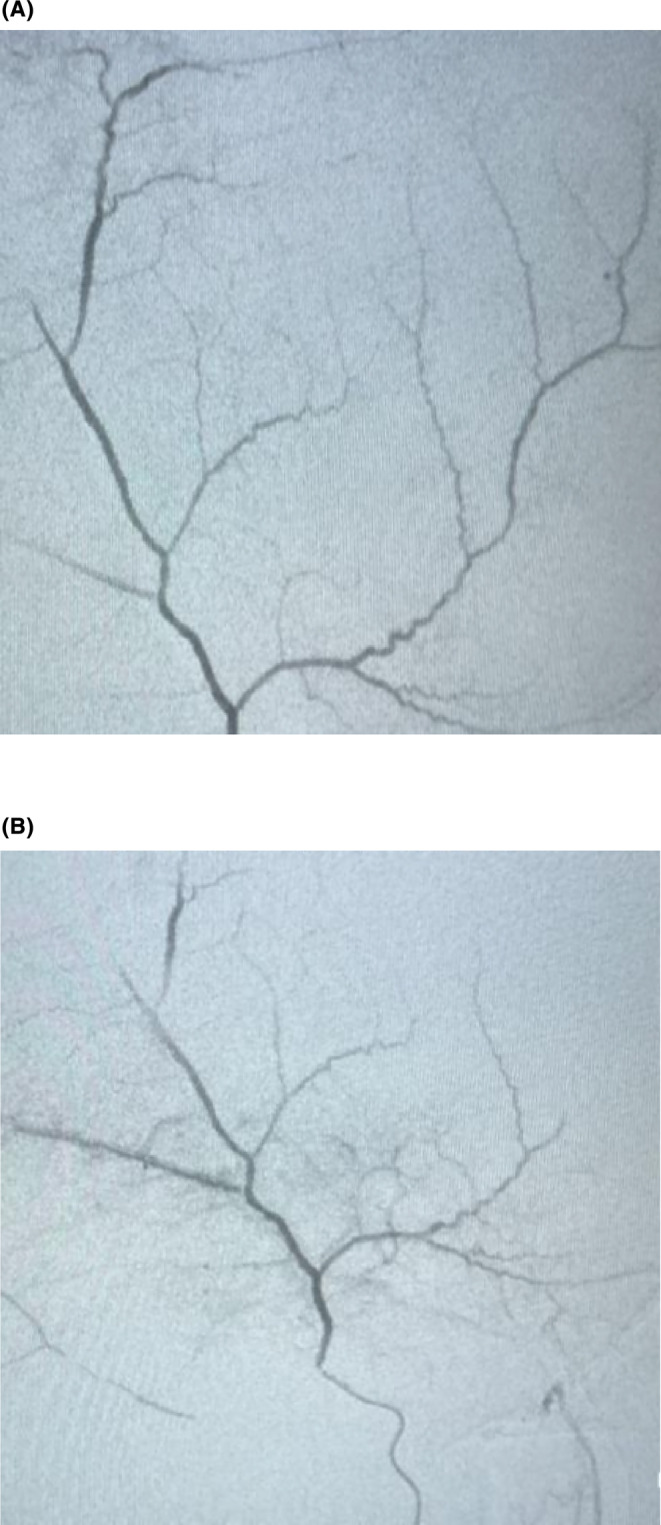
(A) Course of middle meningeal artery before embolization showed on DSA. (B) Middle meningeal artery after embolization showed on DSA.

Subsequently, the decision was made to manage the subdural hematoma through middle meningeal artery embolization (MMAE). Using right femoral artery access, the left‐sided common carotid artery was entered, followed by accessing the external carotid artery. Through the maxillary artery, the middle meningeal artery was reached, and digital subtraction angiography (DSA) of the vessel was performed to identify the specific branch contributing to the subdural hematoma. Selective embolization of this identified branch was then carried out using polyvinyl alcohol (PVA) particles, resulting in a reduced caliber of the middle meningeal artery. (Figure 4A; Figure 4B) Microcatheter embolization was done keeping microcatheter just proximal to the bifurcation of MMA.

**FIGURE 5:**
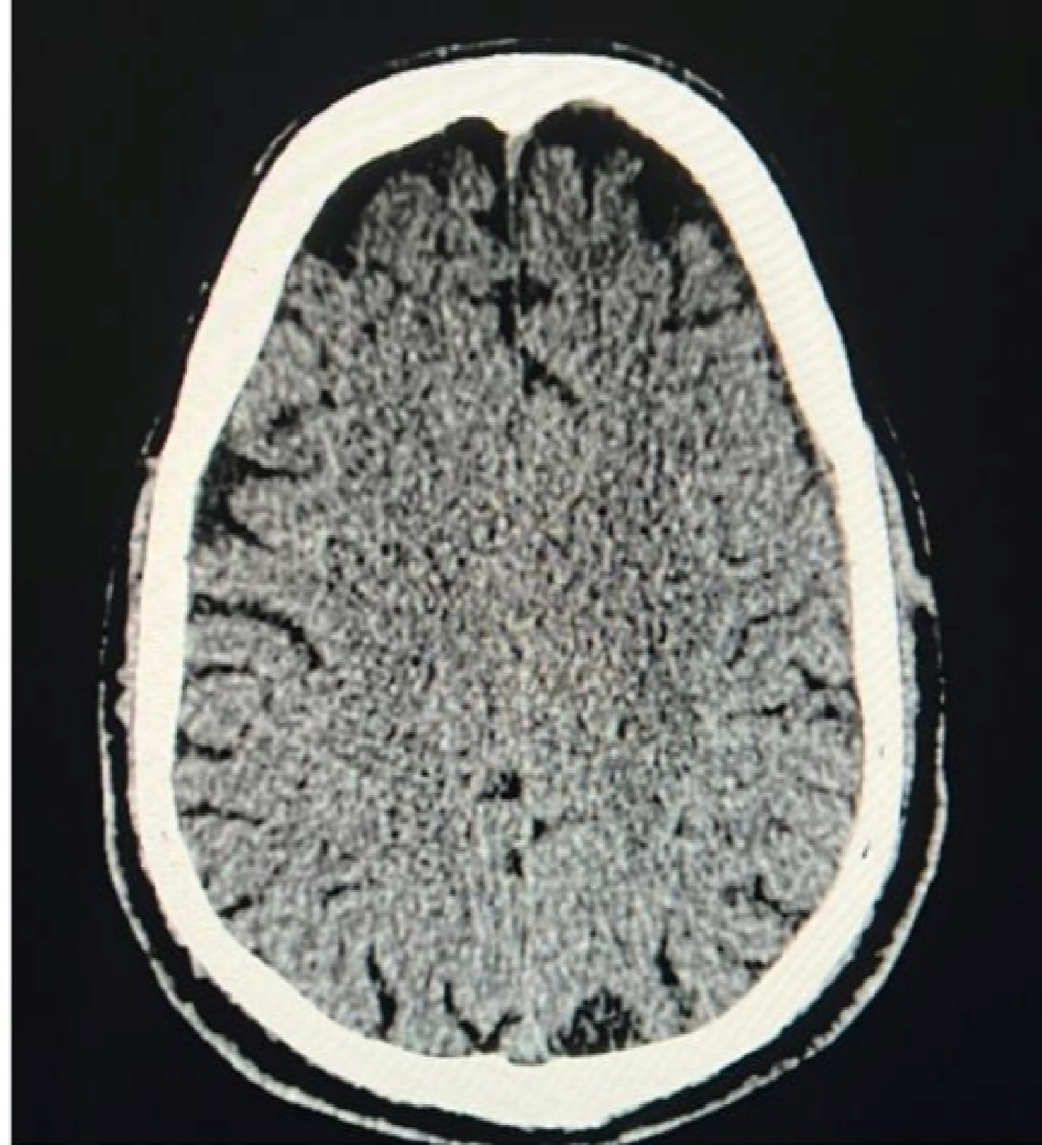
CT scan showing resolution of hematoma after embolization of the middle meningeal artery.

The postoperative course was uneventful, and CT scan demonstrated resolution of the hematoma with no midline shift. (Figure 5).

### 2.4 Follow‐up

At the 3‐month follow‐up, a CT scan revealed no evidence of subdural hematoma, and clinically, the patient reported no complaints.

## 3 DISCUSSION

CSDH frequently manifest in the elderly following minor injuries that do not inflict damage to the underlying brain. Typically, there is a latent period of weeks to months before clinical symptoms become apparent. The peak incidence of CSDH is observed in the sixth and seventh decades of life.^9^ However, our patient, who was in his sixth decade, did not have a history of trauma. Instead, he experienced spontaneous hemorrhage, a recognized cause of CSDH in approximately 10% of cases.^10^

CSDH is often linked to acquired predisposing factors. These factors may include trauma leading to the rupture of bridging veins, the use of antiplatelet medications, coagulopathy arising from liver cirrhosis, and chronic alcohol abuse.^11^, ^12^ In our patient, a thorough examination for predisposing factors was conducted, and none were identified. Therefore, we categorized the condition as idiopathic spontaneous CSDH, recognizing it as one of the causes of CSDH.

The development of CSDH starts with the separation of the dural border cell layer, which triggers healing responses that include dural border cell proliferation, granulation tissue formation, and macrophage deposition.^3^, ^7^, ^13^ Tanaka et al. demonstrated through histological examinations in 1997 that CSDH outer membranes contain three types of vessels—small veins, arteries, and capillaries—that cross the dura mater to connect to the middle meningeal artery (MMA).^14^ Capillary formation in the dura mater contributes to subdural hemorrhage formation, leading to increased hematomas. During this process, the MMA typically appears enlarged.^15^

A single burr‐hole surgery with irrigation and drainage is generally an effective curative treatment for CSDH.^16^ However, up to 20% of patients may experience persistent recurrence of CSDH.^17^ Various surgical methods for recurrent CSDH have been proposed, such as craniotomy to remove the outer membrane, implantation of a reservoir or subdural‐peritoneal shunt, repeated burr‐hole trephination, and endoscopic surgery. The efficacy of these methods remains widely debated, and there is currently no defined set of treatment algorithms for recurrent CSDH.^18^ MMAE has demonstrated a positive therapeutic effect on CSDH and is considered more effective than conventional treatment.^10^

Various materials, including PVA, N‐butyl‐2‐cyanoacrylate (NBCA), coils, and gelatin sponges, can be used in MMA embolization, all demonstrating similar therapeutic outcomes.^19^, ^20^ Takahashi et al. treated three cases of refractory CSDH using embolization via the MMA after several unsuccessful drainage procedures had been performed.^21^ In another study, Tempaku et al. reported five cases of recurrent CSDH successfully treated with MMA embolization, highlighting the usefulness of this interventional procedure.^22^ Orscelik et al. showcased the safety and effectiveness of utilizing adjunct MMA embolization to notably decrease the size and recurrence of CSDH.^23^

The positive outcome observed in our patient, as evidenced by the resolution of the hematoma and absence of complaints at the follow‐up, underscores the efficacy of MMAE as an alternative and effective intervention for challenging and recurrent CSDH cases. This advanced endovascular approach proves valuable in situations where conventional surgical methods may fall short.

## 4 CONCLUSION

For cases of CSDH that prove refractory to conventional burr hole surgery, an alternative and effective management strategy involves the embolization of the middle meningeal artery. This advanced intervention can be successfully executed in facilities equipped with a catheterization laboratory and a skilled team of endovascular specialists. The positive therapeutic outcomes observed in cases treated with MMAE underscore its efficacy as a valuable option in addressing challenging and recurrent CSDH cases.

## AUTHOR CONTRIBUTIONS

**Subash Phuyal:** Conceptualization; data curation; supervision; validation; visualization; writing – original draft; writing – review and editing. **Sushanta Paudel:** Conceptualization; data curation; supervision; validation; visualization; writing – original draft; writing – review and editing. **Suchit Thapa Chhetri:** Conceptualization; data curation; supervision; validation; visualization; writing – original draft; writing – review and editing. **Gopal Sedain:** Conceptualization; data curation; validation; visualization; writing – original draft; writing – review and editing. **Prakash Phuyal:** Data curation; validation; visualization; writing – original draft; writing – review and editing. **Oshan Shrestha:** Supervision; validation; visualization; writing – original draft; writing – review and editing. **Deepa Khanal:** Validation; visualization; writing – original draft; writing – review and editing.

## FUNDING INFORMATION

This article did not receive any grants.

## CONFLICT OF INTEREST STATEMENT

No conflict of interest.

## CONSENT

Written informed consent form was obtained from the patient to publish this report in accordance with the journal's consent policy.

## Data Availability

All the findings are present within the manuscript.
